# CNS critical periods: implications for dystonia and other neurodevelopmental disorders

**DOI:** 10.1172/jci.insight.142483

**Published:** 2021-02-22

**Authors:** Jay Li, Sumin Kim, Samuel S. Pappas, William T. Dauer

**Affiliations:** 1 Medical Scientist Training Program, University of Michigan, Ann Arbor, Michigan, USA.; 2 Cellular and Molecular Biology Graduate Program, University of Michigan, Ann Arbor, Michigan, USA.; 3 Peter O’Donnell Jr. Brain Institute,; 4 Department of Neurology, and; 5 Department of Neuroscience, University of Texas Southwestern Medical Center, Dallas, Texas, USA.

## Abstract

Critical periods are discrete developmental stages when the nervous system is especially sensitive to stimuli that facilitate circuit maturation. The distinctive landscapes assumed by the developing CNS create analogous periods of susceptibility to pathogenic insults and responsiveness to therapy. Here, we review critical periods in nervous system development and disease, with an emphasis on the neurodevelopmental disorder DYT1 dystonia. We highlight clinical and laboratory observations supporting the existence of a critical period during which the DYT1 mutation is uniquely harmful, and the implications for future therapeutic development.

## Introduction

Dystonia manifests as involuntary twisting movements and abnormal postures (1). Dystonia is most commonly caused by insults to basal ganglia structures (especially striatum), including perinatal asphyxia, stroke, and Parkinson disease (2). In these cases, dystonia is termed “secondary,” and there are typically additional neurological signs and symptoms. Dystonia can also occur as an isolated symptom, termed “primary” dystonia (3). The most common inherited primary dystonia is DYT1 dystonia, caused by a loss-of-function (LOF) mutation in the *TOR1A* gene, which encodes torsinA (4). Several features of the natural history of DYT1 dystonia suggest that a critical developmental period is an important feature of disease pathogenesis. The disease is incompletely penetrant, manifesting in approximately one-third of mutation carriers (5). The average age at onset is approximately 12 years, and the great majority of carriers that manifest disease are affected by their teenage years (5). Carriers who do not exhibit dystonia by their early twenties typically remain symptom free for life (5). Considered together, these features suggest that during CNS maturation, there is a unique period of vulnerability to torsinA dysfunction.

Developmental critical periods are discrete windows during normal postnatal maturation when the CNS is uniquely sensitive to certain sensory stimuli (6, 7). During critical periods, heightened plasticity enables defined sensory stimuli to drive circuit formation in ways that support brain function that is adaptive and environmentally appropriate (8–10). Analogous to developmental critical periods, critical periods of vulnerability are discrete windows during postnatal maturation when the CNS is especially vulnerable to certain pathogenic insults, reflecting a unique state of the maturing CNS. These periods of vulnerability are believed to reflect dependence on processes that are strongly upregulated during brain maturation. Enhanced plasticity is one such factor, but other vulnerable processes likely include the myriad events that support maturation, including unique transcriptional programs, morphological changes, and large-scale functional reorganization. Consequently, developmental and vulnerable critical periods are analogous but distinct entities.

Here, we highlight key concepts by briefly reviewing developmental critical periods in normal CNS maturation and critical periods of vulnerability in neurodevelopmental disease. We discuss the significant implications of critical periods of vulnerability for unraveling the pathogenesis of neurodevelopmental disease and conceptual implications for development of therapy. We then highlight these issues through a detailed consideration of DYT1 dystonia as a paradigmatic neurodevelopmental disease.

## Critical periods in normal CNS development

Critical periods are a feature of many processes in healthy neurodevelopment and have been studied extensively for a number of developmental processes (6, 9–12). Principles learned from these studies form the basis for understanding selective windows of vulnerability to pathogenic insults in neurodevelopmental disease.

### Visual system.

A large body of work in multiple species demonstrates that visual input to both eyes during a discrete temporal window is required for normal development of visual function (6, 9, 13–17). There exists a critical period of increased sensitivity to visual deprivation or impairment when such insults can cause irreversible visual dysfunction. Monocular deprivation during the visual critical period in many species causes loss of visual cortical neuron responsiveness to the deprived eye and pallor/reduced size of the lateral geniculate nucleus (13, 15). These changes persist even after the occluded eye is reopened but are attenuated or absent if the monocular deprivation occurs after critical period closure.

The visual critical period can be modified by altering sensory experience. Elimination of essentially all visual stimuli through dark rearing slows the maturation of visual circuits, effectively extending the visual critical period (18, 19). In kittens, visual plasticity, measured as the degree of shifting of visual cortical neuron spiking preference after monocular deprivation, normally rises sharply at 6 weeks of age and declines over the next 10 weeks (19). In contrast, dark-reared kittens maintain a steady increase in plasticity over the first 12 weeks and retain plasticity at 16 weeks of age (19). Dark-reared rats also exhibit a lengthened critical period and heightened plasticity. However this effect is counteracted by environmental enrichment in the form of running wheels and toys, which promote visual system consolidation and maturation (20). After typical critical period closure, 50-day-old rats dark reared from birth without environmental enrichment still exhibit plasticity in response to monocular deprivation, while 50-day-old rats dark-reared from birth with environmental enrichment do not (20). These findings demonstrate that sensory stimuli and experience can influence the critical period, even when the experience involves a modality different from that under study.

Visual system critical periods are directly relevant to human disease. Pediatric cataracts can impair normal visual development (21, 22). Early removal of neonatal cataracts is critical for enabling normal visual function (23). Amblyopia is the failure to develop normal vision, typically because of ocular misalignment. Early therapy (i.e., patching the “good” eye to force use of the at-risk eye) prior to critical period closure is essential. This early intervention improves outcomes and often prevents loss of visual acuity (24–26). These observations highlight the relationship between critical period plasticity, developmental susceptibility, and the capacity for recovery during the critical period.

How do environmental factors such as dark rearing impact the critical period for recovery from monocular deprivation? While our understanding of these events is limited, some work — including genetic, molecular, and pharmacologic manipulations — provides initial insight. Duffy et al. demonstrated that dark rearing increased plasticity in kittens with monocular deprivation (27). In this study, dark rearing appeared to accelerate recovery of visual function through a mechanism that may involve disassembly and reorganization of cytoskeletal elements that limit plasticity (27). The ability of dark rearing to promote recovery in cats was limited to a critical period following monocular deprivation, with effects tapering off by around 6 months of age (28). Studies of the Ly6/neurotoxin 1 (Lynx1) protein highlight a role for nicotinic cholinergic signaling in visual system critical period regulation. Lynx1 binds to and modulates nicotinic acetylcholine receptors (29). Lynx1 expression increases coincident with critical period closure. Monocular deprivation in adult (after critical period closure) WT mice does not induce lasting changes to the visual system, while the same manipulation in adult *Lynx1*-deficient animals shifts visual cortical neuron spiking preference away from the occluded eye (30). *Lynx1*-knockout mice are also able to recover from monocular deprivation at older ages compared with controls. Pharmacological approaches to mimic *Lynx1* deletion by treating WT mice with an acetylcholinesterase inhibitor (thus increasing cholinergic signaling) similarly allowed recovery of visual acuity after monocular deprivation in older animals (30). *Lynx1* deletion and enhancing cholinergic signaling similarly increase plasticity in auditory circuits (31).

The extracellular matrix (ECM) is another established modulator of plasticity. A strong body of work has demonstrated that manipulating specific ECM components influences visual plasticity (32–34). The maturation of ECM into perineuronal nets inhibits axon growth and limits visual plasticity (35). Degradation of chondroitin sulfate proteoglycans, a primary component of perineuronal nets, restores ocular dominance plasticity in mature mice. Taken together, these studies on nicotinic signaling and ECM provide proof-of-concept evidence that genetic, pharmacological, and chemical interventions can alter critical periods. These strategies are valuable means to explore the pathophysiology of neurodevelopmental disease and assess for potential therapeutic effects of critical period modulation.

### Other systems.

Similar to the principles described in the visual system, classic work by Knudsen and colleagues defined critical periods related to barn owl auditory function. These investigators explored the impact of brain maturation on auditory function plasticity by inducing unilateral hearing (by plugging individual ears) at different ages. Animals younger than 8 weeks of age were able to adjust to ear plugging and recovered normal sound localization; however, the ability to adapt to ear plugging was lost at older ages (36). The unique plasticity of younger animals was also demonstrated by their ability to recover normal sound localization after removal of the ear plugs. Ear unplugging prior to 20 weeks of age facilitated rapid recovery of sound localization, but recovery was slower and incomplete when unplugging occurred after 28 weeks of age (37). Visual calibration of auditory space is also developmentally dependent. Shifting the relative position of the owls’ auditory and visual maps (through prismatic goggle wearing) caused inaccurate striking of targets (38). Correction and recalibration of auditory and visual maps at an early age resulted in successful recovery of auditory-visual correlation, but the capacity for recovery was limited to owls younger than 200 days (39). Considered together, these data demonstrate a developmental critical period during which auditory and visual function is strongly modified by sensory experience. Remarkably, this period of plasticity is itself modifiable by experience. Housing the owls in an enriched environment (e.g., in a larger aviary with other owls) essentially eliminated the critical period, enabling recovery of map alignment at all ages tested (39).

Few studies have examined and experimentally manipulated critical periods in motor development. Tail suspension of neonatal and juvenile rats to limit hind limb activity is one approach that has been utilized (40). Suspension between P13 and P31 caused persistent gait abnormalities, leading Walton et al. to label this period as a critical window during which hind limb weight-bearing activity is required for normal motor development (40). Tail suspension earlier or later or for shorter periods of time produced only mild, transient effects. Another approach has been to simulate damage to the corticospinal system by chronically inhibiting primary motor cortex signaling with the GABAergic agonist muscimol. Inactivation of the feline motor cortex from postnatal week 5 to 7 caused inaccurate forelimb reaching, overstepping on a horizontal ladder, and loss of spinal cholinergic interneurons (41). Strikingly, motor function and spinal cholinergic interneuron density were normalized by having the cats perform a forelimb reaching task from postnatal week 8 to 13. In contrast, the reaching task had no benefit if performed from postnatal week 20 to 24 (41). Motor critical periods have not been studied extensively in humans because of ethical considerations regarding experimentation. An extensive body of literature exists documenting a critical period for language acquisition (12). Briefly, while language is a complex process involving sensory, cognitive, and motor processes, the exceptional ability of young children to acquire language skills — which markedly declines by teenage years and beyond — supports the general notion of motor critical periods (42, 43).

Taken together, these studies demonstrate that developmental stage and unique critical period plasticity facilitate normal development. Analogously, in neurodevelopmental disease, the nervous system is likely uniquely susceptible to mutations in genes or other insults that strongly impact the processes that are critical for supporting the enhanced plasticity and other distinctive features of the developing brain.

## Critical periods in neurodevelopmental disease

As with developmental critical periods, critical periods of vulnerability and response to therapy have been investigated for several neurodevelopmental disorders. Here, we consider the disease-related critical periods primarily in the context of gene function in inherited developmental disorders.

Critical periods have been explored in the context of synaptic Ras GTPase-activating protein 1–related (SYNGAP1-related) intellectual disability. Mutations in *SYNGAP1* cause a neurodevelopmental spectrum disorder that includes autism, intellectual disability, and epilepsy (44–46). Studies in mice demonstrate a critical period of vulnerability to *Syngap1* LOF. At P1, deletion of *Syngap1* causes synaptic and cognitive deficits, whereas deletion in adults has no effect (47). Similarly, restoring *Syngap1* expression in adult LOF mice has minimal impact on behavioral phenotypes (47). These data demonstrate a critical period of vulnerability to *Syngap1* impairment during neurodevelopment and a therapeutic critical period after which *Syngap1* supplementation is ineffective.

Angelman syndrome, caused by ubiquitin protein ligase E3A (*UBE3A*) LOF, is a complex neurodevelopmental disease characterized by epilepsy, developmental regression, and autistic features (48). Sonzogni et al. defined a critical period of vulnerability in *Ube3a* LOF mice by demonstrating that embryonic deletion produced robust autism-like phenotypes, but had little effect when depleted in 3- or 12-week-old animals (49). Silva-Santos et al. performed an analogous study to determine whether a therapeutic critical period exists for *Ube3a* restoration and found that the effectiveness of behavioral rescue inversely correlated with age (50). Early (at 3 weeks of age) restoration fully rescued deficits in motor coordination; adolescent (6 week) restoration was partially effective; but adult restoration (14 weeks) was completely ineffective. Prevention of autism-associated phenotypes, such as reduced marble burying and nest building, required embryonic *Ube3a* reactivation, defining a therapeutic critical period (50). These data highlight the pleiotropic effects of pathogenic mutations and the importance of clearly defining the unique processes linked to each symptom.

Not all early-onset neurodevelopmental diseases exhibit critical periods. Mutations in the X-linked gene *MECP2* cause Rett syndrome, which is characterized by an array of neurological symptoms, including ataxia and intellectual disability (51). *Mecp2* deletion in juvenile and adult mice produces similar Rett syndrome–associated behavioral phenotypes and lethality (52). These data are consistent with studies demonstrating that expression of *Mecp2* in adult mice is important for maintenance of neuronal morphology and neuronal networks (53, 54). Indeed, in both adult and juvenile mice, restoration of *Mecp2* expression in a Rett syndrome model effectively suppresses neurological phenotypes (55). A similar scenario has been described in studies of *SHANK3*-related autism. Germline disruption of the autism-linked gene *Shank3* causes synaptic and autism-like behavioral deficits in young mice (56, 57). Strikingly, adult *Shank3* restoration rescued synaptic protein composition, repetitive behaviors, and social interaction deficits (58), but not anxiety or motor performance, which were rescued only when Shank3 was reactivated at juvenile ages (58). These findings reinforce the notion that multiple independent pathophysiological processes can occur in neurodevelopmental disease.

These studies indicate that the responsiveness of the adult nervous system to gene replacement correlates with an ongoing role for the gene in the mature nervous system. In the cases of *Syngap1* and *Ube3a*, adult induction of LOF caused greatly attenuated phenotypes compared with embryonic or juvenile LOF. Analogously, early gene repletion of *Syngap1* or *Ube3a* rescues mouse behavioral phenotypes, but adult gene repletion does not. In contrast, profound phenotypes emerge with adult-onset depletion of *Mecp2*, and genetic restoration in adulthood is effective. These contrasting observations emphasize the importance of determining whether critical periods exist— both for identifying key pathophysiologic processes and for guiding rational therapeutic development.

A striking example of another critical period of vulnerability is that for prenatal alcohol exposure. The selective temporal vulnerability to neurodegeneration caused by ethanol exposure corresponds specifically to the period of rapid synaptogenesis in each brain region (59, 60). Ethanol acts as an NMDA receptor antagonist, and the susceptibility of neurons to NMDA antagonists is similarly developmentally regulated (61). These experiments exemplify how defining selective temporal vulnerability can help to unravel disease pathogenesis and point toward specific therapeutic strategies.

Delayed-onset dystonia due to perinatal hypoxia can occur in humans. Some children who experience perinatal hypoxic injury develop dystonia many years later, typically during their teenage years (62, 63). The delay between the brain injury and the age at dystonia onset suggests that ongoing neurodevelopmental processes may act as a so-called second hit required to trigger symptoms. Work by Aravamuthan et al. indicates that such insults depend upon a critical period to cause dystonia (64). This work demonstrates that perinatal hypoxic injury in rats causes motor and electrophysiologic dysfunction if induced at P7–P8, but not if the hypoxic injury occurs at P5–P6.

A related but distinct issue is relevant in adult-onset disease. Accumulating evidence demonstrates that many disease processes begin long before symptom onset. The study of monogenic disorders with high penetrance, in which presymptomatic mutation carriers can be studied, has been especially informative. For example, fragile X-associated tremor/ataxia syndrome (FXTAS), caused by an intermediate premutation length CGG repeat expansion in the promoter of the *FMR1* gene, is a neurodegenerative disorder that typically manifests after the age of 55, with age-dependent penetrance and progressive clinical features (65–67). Larger expansions of the same repeat cause fragile X syndrome, the most common inherited cause of intellectual disability. Despite the late onset of FXTAS, widespread structural and connectivity changes are observed in younger premutation carriers, long before the emergence of motor or cognitive symptoms (68–70). Similarly, premanifest Huntington disease (HD) is characterized by significant volumetric, microstructural, and functional connectivity changes in brain imaging studies (reviewed in ref. 71). A recent study demonstrated abnormalities in the prenatal cortex of human fetuses carrying the pathogenic HD-related CAG repeat expansion in the *HTT* gene, even though the disease typically manifests decades later (72).

These data suggest that in some cases, the pathogenic cascade of late-onset diseases may begin during brain development. This concept is distinct from that of a classical critical period of sensitivity or vulnerability, but highlights that disruption of developmental pathways may be an important component of the pathogenesis of age-related disease. Work in animal models provides clues to these conceptual differences. Selective expression of mutant *Htt* during development produces movement abnormalities that are analogous to, but less severe than, those in mice harboring mutant *Htt* their entire lives (73). These data suggest that HD is not characterized by a critical period, but that a potentially *HTT*-independent pathogenic cascade begins much earlier than symptom onset. In both cases, it is important to identify early disease processes that are likely more amenable to gene therapy prior to onset of potentially irreversible damage.

Even Alzheimer disease, the paradigmatic age-related neurodegenerative illness, is now understood to begin decades prior to symptom onset (74–78), which may in part explain the failures of disease-modifying therapies (79–81). These observations highlight that defining the earliest manifestations of disease pathophysiology, including those emerging during brain development, is likely essential to developing efficacious therapies for a larger group of diseases than those with an obvious developmental component.

## DYT1 dystonia and torsinA

DYT1 dystonia is caused by an in-frame *TOR1A* deletion mutation that results in removal of a single glutamic acid residue (ΔE) from the torsinA protein (4). The expression pattern of torsinA in mice is consistent with an important role in neurodevelopment. TorsinA is expressed at higher levels in neural compared with nonneural tissues (82, 83), and striatal torsinA expression is greater in developing than mature mice (84). Limited data exist documenting the expression pattern of torsinA in human postmortem brains (85), but they suggest that it is expressed by the early postnatal period.

Multiple lines of evidence suggest that the ΔE mutation impairs torsinA function. TorsinA is an AAA+ ATPase localized to the nuclear envelope (NE)/endoplasmic reticulum endomembrane space. It requires the cofactor lamina-associated polypeptide 1 (LAP1) or luminal domain–like LAP1 (LULL1) for ATPase activity; the ΔE mutation disrupts interaction with these cofactors, impairing ATPase activity (86–90). ΔE torsinA accumulates aberrantly at the NE and can recruit WT torsinA to this structure. This finding is consistent with a dominant-negative molecular mechanism and a role at the NE.

Mouse genetic studies provide in vivo data confirming that the ΔE mutation impairs torsinA function. *Tor1a^–/–^, Tor1a*^ΔE/–^*,* and *Tor1a*^Δ*E/Δ*E^** mice are phenotypically similar: each of these genotypes exhibits early postnatal lethality and morphologic abnormalities of the neuronal nuclear membrane known as “blebs” (91). These studies define ΔE torsinA as a LOF allele. Additional in vivo work confirms the LOF effect of the ΔE mutation, but demonstrates that ΔE torsinA retains some function (i.e., is a hypomorphic allele). For example, conditional deletion of *Tor1a* from the CNS (Nestin-Cre; *Tor1a^fl/–^*) causes neurodegeneration in a discrete subset of sensorimotor regions, including cortex, thalamus, red nucleus, facial nerve nucleus (7N), and cerebellum (92). Mice made to selectively express the *Tor1a*^ΔE^** allele in the CNS (Nestin-Cre; *Tor1a*^fl/*Δ*E^**) exhibit the same pattern but significantly less cell loss than *Tor1a*-deficient mice, indicating some residual torsinA function. Consistent with this conclusion, Nestin-Cre; *Tor1a^fl/–^* mice die by approximately P16, whereas Nestin-Cre; *Tor1a*^fl/*Δ*E^** mice exhibit delayed postnatal growth and overtly abnormal postures, but not early lethality (92). A gene dosage study further supports the conclusion that *Tor1a*^ΔE^** is a hypomorphic LOF allele (93).

**Figure 1:**
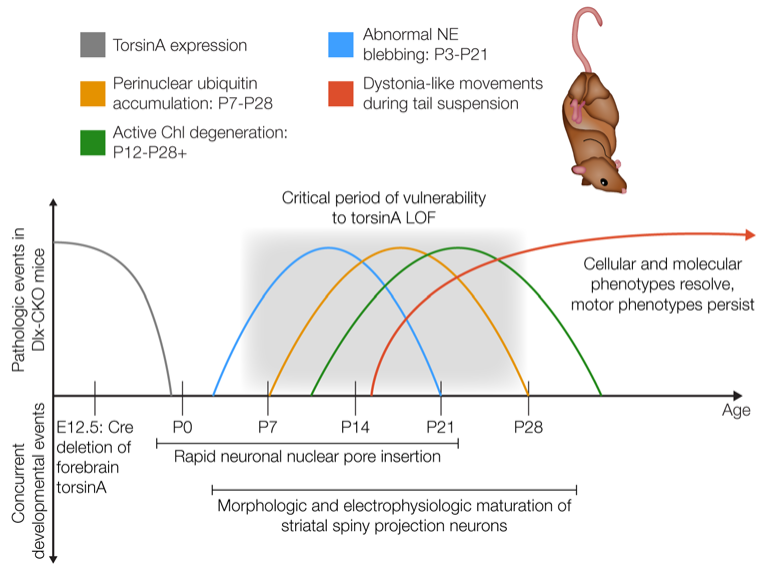
A neurodevelopmental model of DYT1 pathogenesis. Timeline of molecular, cellular, and behavioral events in Dlx-CKO mice, as well as concurrent and potentially related developmental processes (109, 140). Molecular and cellular phenotypes emerge throughout a discrete period of vulnerability to torsinA impairment during early postnatal CNS maturation. These phenotypes rapidly disappear or stabilize, but motor dysfunction persists for life. This model highlights that initial torsinA-linked molecular events can lead to permanent circuit dysfunction delinked from the initial genetic insult. The sources for the phenotypes defined are as follows: NE blebbing (84), perinuclear ubiquitin accumulation, cholinergic interneuron (ChI) degeneration, and motor dysfunction (94). Illustrated by Rachel Davidowitz.

Modeling DYT1 dystonia with torsinA LOF in mice demonstrates a critical role for torsinA in early postnatal brain maturation. The phenotypes described above for mice with conditional *Tor1a*^ΔE/–^** CNS mutations are not present at birth, emerge in the first 1–2 weeks of life, and do not subsequently progress (92). Deletion of *Tor1a* from all cerebellar cells (using *En1-Cre*) causes almost complete loss of deep cerebellar neurons within the first 2 weeks of life, but no further change up to 1.5 years of age (93). Conditional *Tor1a* deletion in forebrain of mice (using *Dlx5/6-Cre*; herein referred to as Dlx-CKO) causes a highly selective loss of dorsal striatal cholinergic interneurons that begins at approximately P12 and appears complete by approximately P30 (Table 1 and Figure 1; ref. 94). Assessment of human postmortem striatal tissue from patients with DYT1 dystonia shows a selective cholinergic abnormality (94), providing support for the validity of the model. Motor symptoms in the Dlx-CKO model emerge in juvenile mice (~P15) and are responsive to clinically used drugs, indicating shared pathophysiology with DYT1 dystonia (94). These data highlight a potential critical period of vulnerability in the first few postnatal weeks, when neurodegeneration is ongoing and motor symptoms begin (Figure 1). Interestingly, this time period in rodents appears to roughly correlate with the preadolescent period in humans (95), when symptom onset in DYT1 dystonia typically occurs (5).

Unique developmental effects are also observed in models of DYT6 dystonia, which is caused by mutations in the *THAP1* gene (96). As in DYT1 dystonia, symptom onset in DYT6 dystonia typically emerges during childhood and is incompletely penetrant (97). A large number of dominantly inherited *THAP1* mutations cause DYT6 dystonia, including early truncations, consistent with a LOF mechanism. Mice with CNS-specific *Thap1* mutations exhibit impaired myelination and motor dysfunction as juveniles (98). Myelination largely normalizes in adulthood, but motor dysfunction persists (98). THAP1 is therefore essential for early postnatal myelination but appears largely dispensable in adulthood. These data indicate a model whereby a transient developmental defect can resolve but nevertheless causes permanent CNS dysfunction.

Studies in DYT1 models have documented aberrant corticostriatal plasticity (99, 100) and suggest a role for striatal cholinergic interneurons in this phenotype (99, 101–103). Augmented and premature long-term potentiation at corticostriatal synapses is observed in immature *Tor1a*^ΔE/+^** mice (Table 1; ref. 104). Alterations in plasticity correlated with increased brain-derived neurotrophic factor (BDNF) levels, which were observed in juvenile but not adult *Tor1a*^ΔE/+^** striatum. BDNF antagonism rescued plasticity deficits in juvenile, but not adult, mice (104). These findings are consistent with those described above, indicating a developmental window.

One study, employing RNAi-mediated knockdown rather than gene deletion of torsinA, proposes an opposite model of DYT1 pathophysiology (105). The authors argue that developmental compensation prevents early torsinA LOF phenotypes, and that acute knockdown at later ages is required in order to model the disease. However, the multiple examples of early postnatal phenotypes in the *Tor1a* mutants described above and the fact that the human disease itself is caused by a germline mutation are not consistent with this model. RNAi is known to exert toxic off-target effects (106), and the effects of gene knockdown can differ considerably from those of traditional gene-targeting strategies (107), suggesting alternative explanations for these acute knockdown findings.

Molecular and cellular studies of torsinA provide clues to its essential role during postnatal maturation. Several studies demonstrate that torsinA functions at the NE. Postmigratory neurons from *Tor1a^–/–^* and *Tor1a*^Δ*E/Δ*E^** mice exhibit abnormal blebbing of the inner nuclear membrane (INM), forming Ω-shaped outpouchings (blebs) that are connected to the INM by a neck-like structure (83, 91, 108). These structures are not observed in any other cell types or in migrating neurons. NE blebs emerge in a caudal-rostral gradient reflective of CNS development. In newborn mice, earlier-maturing caudal regions exhibit higher percentages of nuclei with NE blebs than later-maturing rostral regions. NE blebs appear to be linked to nuclear pore complex (NPC) biogenesis. Maturing neurons dramatically increase the number of NPCs during a discrete developmental window (109) that likely corresponds to a demand for increased transcription and translation as these cells mature and integrate into circuits. NE blebs appear similar to an intermediate structure of interphase NPC biogenesis (108, 110, 111) and contain NPC components (108, 112). TorsinA-null neurons exhibit mislocalized, incomplete-appearing NPCs that contain early-added nucleoporins but lack components that are added later in the process (110, 113), consistent with a halted intermediate structure. Furthermore, depleting an essential component of NPC assembly prevents formation of NE blebs in a cellular model of torsin LOF (112). The period of rapid NPC biogenesis could represent a strongly upregulated developmental process that increases demand on torsinA function, rendering the developing CNS uniquely vulnerable to torsinA LOF.

**Figure 2:**
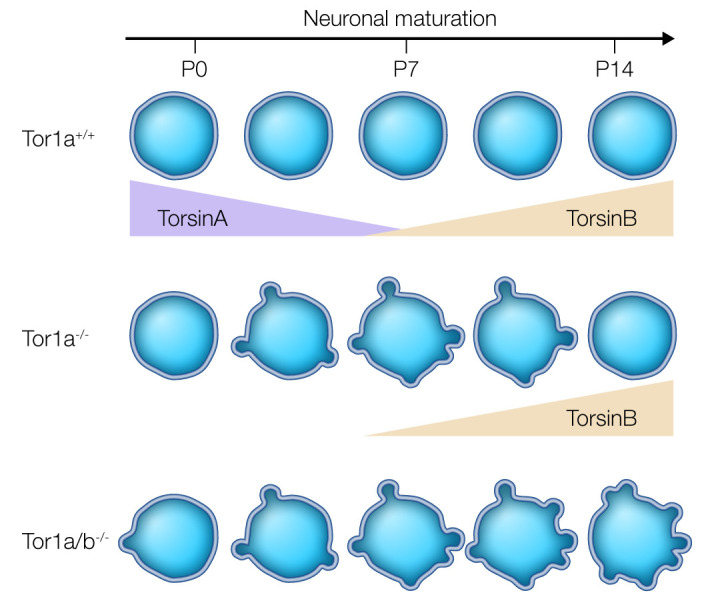
TorsinB dictates developmental vulnerability to torsinA LOF–related NE blebbing. Abnormal NE blebbing occurs during a developmental window when torsinB levels are low. Blebs resolve as torsinB levels rise during maturation. Simultaneous deletion of torsinA and torsinB prevents bleb resolution, highlighting how the torsinB expression level dictates susceptibility to torsinA LOF. Illustrated by Rachel Davidowitz.

Another factor that influences vulnerability of the developing CNS to torsinA LOF is the expression level of the torsinA paralog torsinB. TorsinB shares sequence similarity (approximately 68%) with torsinA and is activated by the same cofactors that activate torsinA catalytic function (88). TorsinB levels in the striatum begin to increase in the second postnatal week, and torsinA levels decrease during the same period (Table 1 and Figure 2; ref. 84). The increase in torsinB expression correlates with resolution of NE blebs (Figure 2), suggesting that this upregulation may contribute to the normalization of the torsinA-related phenomenon. Consistent with this possibility, deleting both *Tor1a* and *Tor1b* prevents bleb resolution, and torsinB overexpression suppresses NE bleb formation in vitro (Figure 2; ref. 84). These data highlight how the relative levels of torsinA and torsinB that characterize normal development may correspond to a critical period when torsinA function is essential.

TorsinB expression levels also modulate behavioral abnormalities and neurodegeneration caused by torsinA LOF. Homozygous germline deletion of torsinB appears phenotypically silent (84, 114). In DYT1 mouse models, however, reducing torsinB levels worsens behavioral and neuropathological phenotypes, whereas increasing torsinB expression completely rescues abnormal movements and neuronal loss (114). *Emx1-Cre*–mediated deletion of torsinA in mice causes cortical thinning and motor dysfunction; simultaneous deletion of torsinA and torsinB in the same spatial field exacerbates both neurodegeneration and motor dysfunction in all mice. Conversely, torsinB overexpression in multiple torsinA-CKO mouse models is protective, preventing phenotypes including early lethality, neurodegeneration, and motor dysfunction (114). Critically, torsinB overexpression also rescues the neuropathological and behavioral features of mice that selectively express the *Tor1a*^ΔE^** allele in the CNS (Nestin-Cre; *Tor1a*^fl/*Δ*E^**), establishing the effectiveness of this intervention in the context of pathogenic ΔE torsinA. These studies provide further evidence that developmental changes in torsinB expression may play a key role in dictating the critical period of vulnerability to torsinA LOF, and that modulating torsinB levels may be an effective therapeutic strategy for DYT1 dystonia.

While DYT1 and some other genetic forms of dystonia manifest primarily in childhood, most cases of sporadic dystonia occur in adulthood (115). Why do some forms of dystonia display a developmental vulnerability and onset while others do not? The mechanisms of adult-onset primary dystonia are poorly understood because nearly all cases are idiopathic. DYT25 dystonia, caused by dominant mutations in *GNAL*, is the single inherited form of dystonia that manifests primarily (although not exclusively) during adulthood (3). *GNAL* encodes the α (stimulatory) subunit of the Gα(olf) heterotrimeric complex that participates in D1 receptor signaling (116). Dystonia arising from multiple etiologies has been linked to a set of common downstream abnormalities, including altered basal ganglia output, reduced cortical inhibition, impaired sensorimotor integration, and maladaptive plasticity (117–126). Interestingly, in some patients DYT25 dystonia manifests during childhood, and GNAL participates in several of the processes affected by genes causing childhood-onset dystonia. Considered together, these facts suggest that diverse molecular processes converge on one or more dystonia-related circuit mechanisms, but that the developmental susceptibility to these mutations likely depends on the specific gene and its role, or lack thereof, in developmental processes.

## Clinical implications and future directions

Several central themes emerge in the consideration of strategies for neurodevelopmental diseases. The effectiveness of etiologically based molecular therapies for many (and likely most) neurodevelopmental disorders will be restricted to periods when the developmental process(es) directly or closely linked to disease are initially active, prior to downstream circuit reorganization or neurodegeneration. In contrast, following symptom onset (especially in established disease), circuit-based interventions will typically be required. While caution is warranted in extrapolating from mouse models of disease, available data indicate, for example, that basal ganglia output is unlikely to change following torsinA repletion in adulthood, whereas patients with DYT1 and other etiologic forms of dystonia with long-standing symptoms benefit from the circuit-based therapy of deep brain stimulation (124, 127).

For DYT1 dystonia, future studies exploring a therapeutic critical period for *Tor1a* gene replacement will require novel genetic tools for reactivation and/or supplementation of torsinA expression in DYT1 dystonia model systems at different developmental stages. In vivo torsinA rescue studies in symptomatic animal models of disease are important for advancing understanding of DYT1 pathogenesis in relation to developmental processes and assessing the therapeutic potential (and timing requirements) of torsinA augmentation. If torsinA function is especially important during early postnatal maturation, as suggested by the work discussed above, then restoration strategies will need to occur before torsinA-resistant downstream consequences ensue. Genetic screening can identify mutation carriers, but intervention is unlikely to be feasible in presymptomatic children with an incompletely penetrant disease. These considerations highlight the importance of identifying early biomarkers, perhaps through imaging, that can accurately predict which carriers are destined to develop dystonia. The only known factor that impacts penetrance is the torsinA D216H substitution, which is protective when present in *trans* to the ΔE torsinA allele (128). In addition to defining the temporal constraints of torsinA repletion, future studies should be directed at identifying other factors that influence penetrance.

An alternative to torsinA-based strategies is torsinB augmentation. An advantage of this therapeutic target is that, in contrast to torsinA-based strategies, it will not increase levels of pathogenic ΔE torsinA. TorsinB expression levels determine the tissue specificity (82, 83) and temporal pattern (84) of abnormal NE blebbing, and torsinB overexpression rescues the behavioral and neurodegenerative consequences of torsinA LOF (114). These observations provide proof-of-concept evidence that torsinB augmentation could potentially suppress or prevent dystonia in patients with DYT1 dystonia. TorsinB likely exerts its beneficial effects through pathways overlapping with torsinA, so any temporal constraints for torsinA-mediated rescue will also likely apply to torsinB supplementation. The efficacy of torsinB rescue in reversing motor dysfunction in later-stage (already symptomatic) mice has not been tested.

A potential approach to increasing receptiveness to etiologically based (genetic) therapies is upregulation of the relevant critical period pathways. As reviewed above, restoration of critical period plasticity, using pharmacologic, genetic (30, 31), and/or environmental manipulations (20, 129), has been performed in a variety of contexts. It may be possible to increase or extend responsiveness to gene replacement in DYT1 dystonia and other developmental diseases. However, this approach in DYT1 dystonia requires a more complete understanding of torsinA function as well as the molecular and cellular correlates of torsinA LOF in the human nervous system. Importantly, evidence for aberrant plasticity has been suggested in clinical (117, 120, 130, 131) and laboratory studies (100, 104). Altering plasticity or attempting to restore juvenile-like plasticity could exacerbate symptoms. Similar considerations pertain to strategies to diminish plasticity during disease onset.

An alternative to either intervention during critical period or modifying/recreating critical period–like plasticity is development of therapeutic approaches that are not dependent on a specific developmental landscape for efficacy. Available therapies for primary dystonia, including anticholinergic drugs and altering basal ganglia output (e.g., pallidotomy or deep brain stimulation) (123, 132), already target circuit dysfunction downstream of the genetic insult. It is essential to develop and refine treatment strategies to reduce off-target effects and increase efficacy, especially for those with long- standing, stable disease.

One promising area of investigation is targeting of striatal cholinergic dysfunction. Several lines of evidence implicate the striatum as a key node in dystonia pathogenesis (2, 124, 133–135) and directly implicate striatal cholinergic interneurons as a cell type relevant to DYT1 pathophysiology. Striatal cholinergic interneurons contribute to the aberrant corticostriatal plasticity observed in DYT1 mice (99, 100, 136, 137). Antimuscarinics and other selective muscarinic agents can correct aberrant plasticity in DYT1 mice (99, 102). Within the striatum, cholinergic interneurons are uniquely vulnerable to torsinA LOF, as a subset of these neurons degenerate in the absence of torsinA (94, 138). The degeneration of these neurons is coincident with dystonia-like motor symptoms in multiple mouse models (94, 139), and rescue of these neurons correlates with motor symptom suppression (114, 139). These observations suggest that striatal cholinergic interneuron dysfunction may contribute to the expression of motor symptoms.

A more detailed understanding of cholinergic interneuron vulnerability to torsinA LOF and the nature of the contribution of their aberrant function to DYT1 dystonia could provide new concepts for therapeutic development before and after a critical period. Understanding the initial vulnerability of these neurons will be relevant to preventing or suppressing critical period events. Defining the subsequent dysfunctional state of these cells could provide a path forward for the treatment of later-stage, established dystonia. Expanding on studies involving correction of plasticity (99, 102–104) to determine whether muscarinic or nicotinic agents that act more selectively can suppress motor dysfunction in symptomatic models of DYT1 dystonia may be a promising direction. More fundamentally, the role of striatal cholinergic signaling in motor learning, and how these mechanisms may go awry in dystonia, needs considerable clarification. Because such studies would target circuit mechanisms proximate to the motor symptoms, they may benefit treatment of multiple forms of dystonia. A multifaceted approach is necessary to develop a range of therapeutic strategies to effectively combat dystonia symptoms of any etiology and at all stages of disease.

## Figures and Tables

**Table 1:**
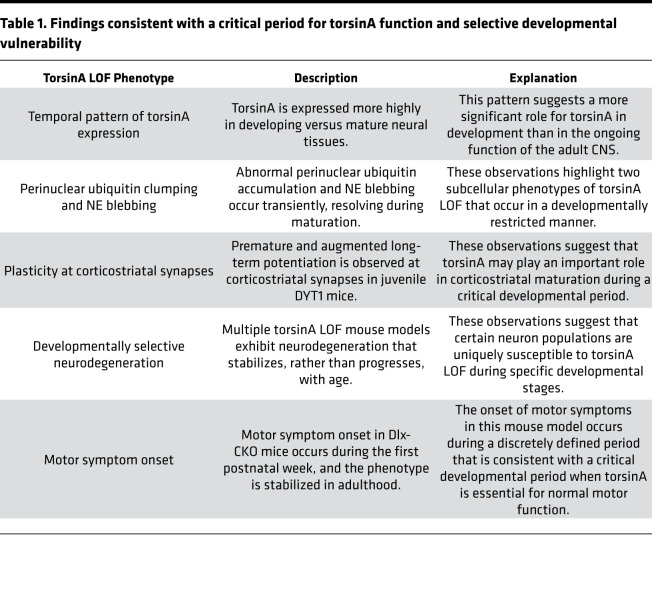
Findings consistent with a critical period for torsinA function and selective developmental vulnerability

## Version 1 02/22/2021

Electronic publication
